# Mental representations of speech and musical pitch contours reveal a diversity of profiles in autism spectrum disorder

**DOI:** 10.1177/13623613221111207

**Published:** 2022-07-18

**Authors:** Li Wang, Jia Hoong Ong, Emmanuel Ponsot, Qingqi Hou, Cunmei Jiang, Fang Liu

**Affiliations:** 1 University of Reading, UK; 2 The Chinese University of Hong Kong, Hong Kong; 3 Ghent University, Belgium; 4 Nanjing Normal University of Special Education, China; 5 Shanghai Normal University, China

**Keywords:** autism spectrum disorder, mental representation, music, pitch processing, reverse correlation, speech

## Abstract

As an information-bearing auditory attribute of sound, pitch plays a crucial role in the perception of speech and music. Studies examining pitch processing in autism spectrum disorder have produced equivocal results. To understand this discrepancy from a mechanistic perspective, we used a novel data-driven method, the reverse-correlation paradigm, to explore whether the equivocal findings in autism spectrum disorder have high-level origins in top–down comparisons of internal mental representations of pitch contours. Thirty-two Mandarin-speaking autistic individuals and 32 non-autistic individuals undertook three subtasks testing mental representations of pitch contours in speech, complex tone and melody, respectively. The results indicate that while the two groups exhibited similar representations of pitch contours across the three conditions, the autistic group showed a significantly higher intra-group variability than the non-autistic group. In addition, the two groups did not differ significantly in internal noise, a measure of the robustness of participant responses to external variability, suggesting that the present findings translate genuinely qualitative differences and similarities between groups in pitch processing. These findings uncover for the first time that pitch patterns in speech and music are mentally represented in a similar manner in autistic and non-autistic individuals, through domain-general top–down mechanisms.

**Lay abstract:**

As a key auditory attribute of sounds, pitch is ubiquitous in our everyday listening experience involving language, music and environmental sounds. Given its critical role in auditory processing related to communication, numerous studies have investigated pitch processing in autism spectrum disorder. However, the findings have been mixed, reporting either enhanced, typical or impaired performance among autistic individuals. By investigating top–down comparisons of internal mental representations of pitch contours in speech and music, this study shows for the first time that, while autistic individuals exhibit diverse profiles of pitch processing compared to non-autistic individuals, their mental representations of pitch contours are typical across domains. These findings suggest that pitch-processing mechanisms are shared across domains in autism spectrum disorder and provide theoretical implications for using music to improve speech for those autistic individuals who have language problems.

## Introduction

Autism spectrum disorder (ASD) is a neurodevelopmental disorder defined by (1) atypical development in the areas of social communication and social interaction and (2) restricted and repetitive patterns of behaviours and interests (American Psychiatric Association, 2013). ASD is typically diagnosed in childhood and has a wide range of symptoms, each of which may differ in their severity across different individuals. One instance of this is communication: some autistic children communicate verbally, some non-verbally, and others a combination of both (e.g. they have very few words and may supplement their verbal communication using specialist language assistance software or the Picture Exchange Communication System). Impairments in language and communication may have varying manifestations (Eigsti et al., 2011), but critically, far from impairment, many autistic individuals demonstrate exceptional musical abilities, including extraordinary musical memory and increased sensitivity to musical pitch (Heaton, 2009).

The dissociable ability to process language and music in some autistic individuals has attracted considerable attention from researchers in an attempt to understand whether this dissociation is a general characteristic of the ASD population (Jiang et al., 2015; Lai et al., 2012; Sharda et al., 2015). The findings of whether autistic individuals show impaired speech but enhanced music processing are particularly relevant to an ongoing debate about whether speech and music share the same underlying processing systems (Albouy et al., 2020; Norman-Haignere et al., 2015; Zatorre & Gandour, 2008). Some researchers have proposed a modular or domain-specific framework (Fodor, 1983, 2001; Peretz, 2009; Peretz & Coltheart, 2003; Peretz et al., 2015; Peretz & Zatorre, 2005), emphasizing that music processing utilizes modules that are not shared with speech processing. Others have suggested that there are shared or domain-general mechanisms underlying the processing of information across both domains (Koelsch, 2011; Koelsch & Siebel, 2005; Patel, 2008; Sammler et al., 2009). Given that there may be differences in specialized representations and commonalities in basic processing mechanisms between music and speech (Patel, 2008), the investigation of top–down comparisons of internal mental representations of speech and music in ASD will not only help better understand the underlying processing systems across domains (Ouimet et al., 2012), but also offer theoretical implications for using alternative options to remediate possible difficulties in either domain, such as using music-related activities to improve language production and comprehension in ASD (Williams et al., 2021).

**Figure 1.**
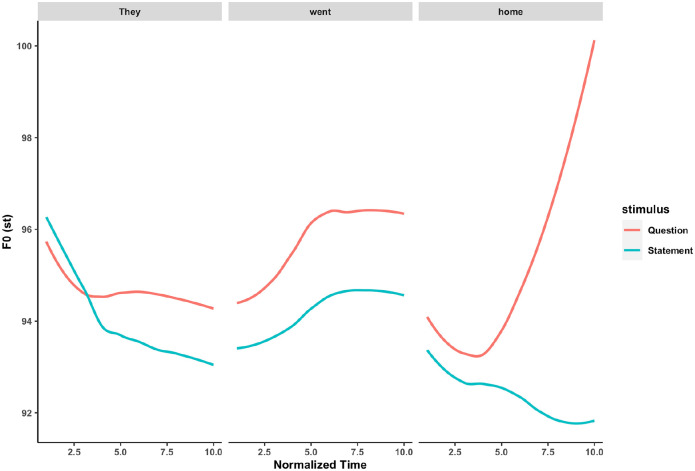
Time-normalized pitch contours of ‘They went home’, spoken either as a statement or as a question.

**Figure 2.**
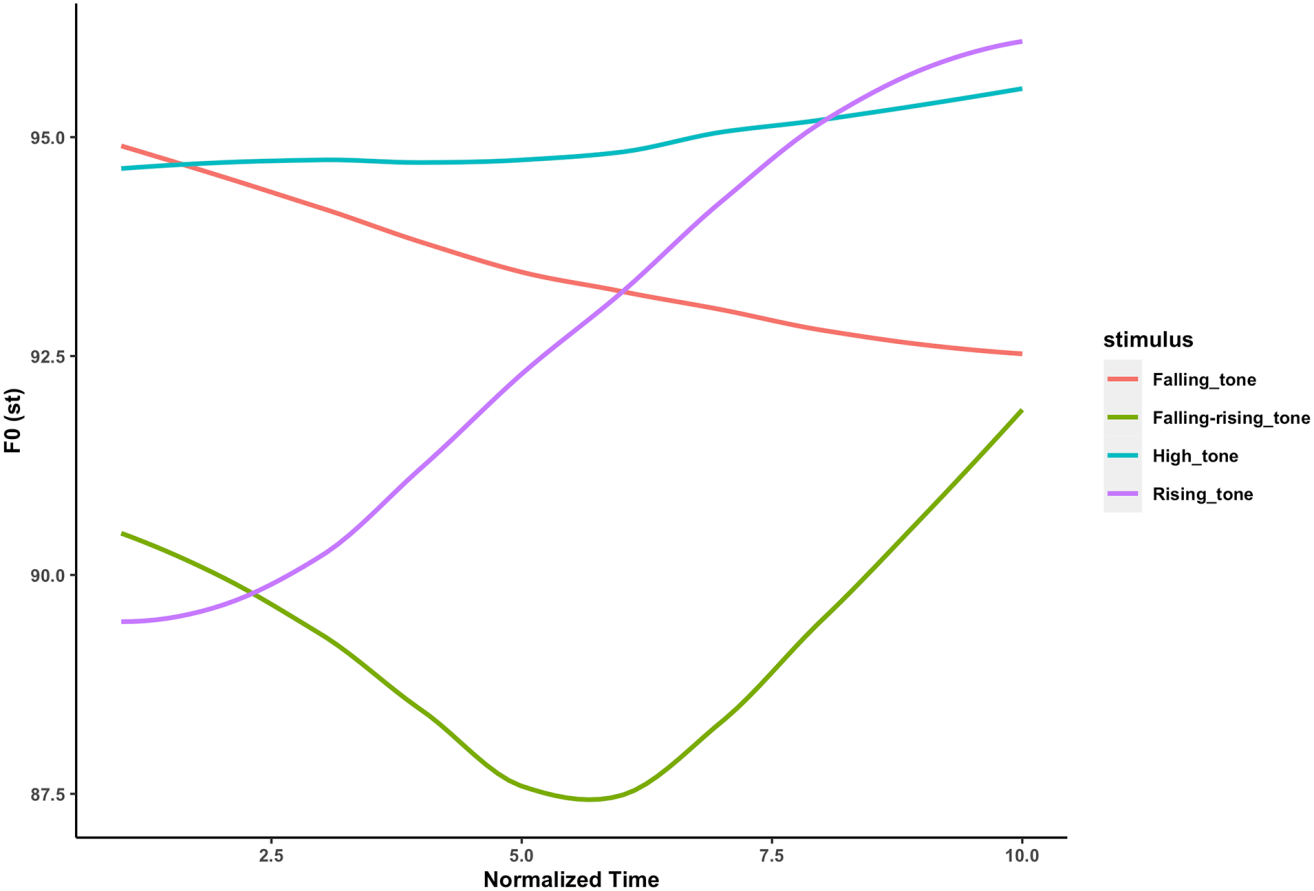
Time-normalized pitch contours of the four Mandarin tones on the syllable /mi/.

Pitch, as a salient acoustic feature shared between language and music, provides a natural laboratory for comparative studies of the two domains. Specifically, pitch is not only a key auditory attribute for the processing of melodies and chords in music (Krumhansl, 2004; Sadakata et al., 2020), but also a crucial cue in delivering prosodic meaning in speech. For example, questions are usually associated with a rising pitch contour (e.g. ‘They went home?’), whereas statements are associated with a falling pitch contour (e.g. ‘They went home’.) (Bidelman et al., 2011; also see Figure 1). In tone languages like Mandarin, pitch contours are also used to differentiate semantic meaning at the word level (Klein et al., 2001), such that the same syllable /mi/ could mean ‘to squint’ (‘眯’), ‘lost’ (‘迷’), ‘rice’ (‘米’) and ‘honey’ (‘蜜’), with a high tone, a rising tone, a falling–rising tone and a falling tone, respectively (See Figure 2).

Regarding pitch processing in ASD, most studies, especially the earlier ones, have suggested that autistic individuals exhibit exceptional musical pitch sensitivity compared with their non-autistic counterparts (O’Connor, 2012; Ouimet et al., 2012). For example, autistic individuals show enhanced abilities to discriminate (e.g. same or different) and identify (e.g. low or high) pitch in pure tones (Bonnel et al., 2003, 2010; Heaton et al., 1998; O’Riordan & Passetti, 2006). Beyond these simple stimuli, enhanced pitch processing in ASD has also been observed in musical melodies, including identification of melodic pitch contour (Jiang et al., 2015) and detection of a local pitch change in a melody (Heaton, 2005; Heaton et al., 1999; Mottron et al., 2000; Stanutz et al., 2014). Therefore, enhanced musical pitch processing has been viewed as a characteristic of many autistic individuals. Nevertheless, typical or even impaired musical pitch processing has also been reported in the literature (Cheng et al., 2017; Germain et al., 2019; Heaton, Williams, et al., 2008; Jamey et al., 2019; Jones et al., 2009; Kargas et al., 2015; Schelinski et al., 2017). For instance, more recent studies investigating pitch discrimination between two tones (e.g. which one is higher) found that after controlling for age and IQ, autistic individuals performed either similarly to (Jones et al., 2009) or worse than non-autistic individuals (Kargas et al., 2015) at the group level. Still, enhanced pitch discrimination was found in a subgroup of autistic participants, for example, 20% in Jones et al. (2009) and 9% in Kargas et al. (2015). Hence, while enhanced musical pitch processing has been observed in ASD, it may only be evident among a subgroup of autistic individuals.

In contrast to musical pitch processing, pitch-mediated speech-processing ability is typically viewed as a skill that autistic individuals have difficulty with, especially prosodic and semantic pitch processing, including identifying and discriminating questions and statements (Jiang et al., 2015), distinguishing lexical stress contrasts (Paul et al., 2005), as well as encoding lexical tones (Lau et al., 2021; Wang et al., 2017). Given that semantic and prosodic information play a crucial role in speech communication and interaction, this atypical pitch processing in speech may hinder language acquisition and development in ASD (Schreibman et al., 1986). However, some studies showed enhanced identification of pitch contours (e.g. rising, falling, rising–falling, falling–rising) and discrimination of pitch differences (e.g. are these two sounds the same?) across speech and musical conditions, suggesting that superior pitch processing in autistic individuals is not limited to music but also extends to speech (Heaton, Hudry, et al., 2008; Järvinen-Pasley et al., 2008; Järvinen-Pasley & Heaton, 2007). However, a recent study suggested similar performance on identification and discrimination of statements and questions between autistic and non-autistic individuals across different age cohorts (Wang, Beaman, et al., 2021).

In summary, although pitch processing in ASD has been studied widely across music and speech, the findings are mixed and pointing towards all possible directions, since enhanced, intact or impaired pitch processing has all been reported across domains. It is important to acknowledge that some discrepancy in findings may be inherent to sampling variability as well as sampling size, considering that ASD is a heterogeneous group and a high variability in the ASD sample has been found in many areas, including pitch processing (Kargas et al., 2015; Milne, 2011; Valla & Belmonte, 2013). However, these apparently contradictory findings can be further explained by considering the complex and multi-stage aspects of pitch processing (Germain et al., 2019; Haesen et al., 2011), whose facets may be differently recruited and exposed depending on task demands, thus leading to various conclusions about the impact of ASD on pitch processing across studies. Specifically, a wide range of stimuli (e.g. from pure tone to natural speech utterance or musical melody) and task complexity (e.g. from simple tone discrimination to local temporal deviant analysis in complex sequences) have been explored in pitch-processing studies in ASD, with a fundamental question remaining unresolved; that is, why low-level pitch sensitivity can be mismatched with performance on musical or linguistic pitch-processing tasks (Jiang et al., 2015; Lau et al., 2021; Paul et al., 2005; Wang et al., 2017). We hypothesized that the mismatched extrapolations may have higher-level origins, given that in contrast to bottom–up sensory processing for simple detection tasks, discriminating complex speech or musical sequences recruit top–down processing (Germain et al., 2019; Haesen et al., 2011). In particular, autistic individuals may have either limited or distorted top–down comparisons of internal mental representations of time-varying pitch contours with incoming natural, complex signals, resulting in an impaired ability to compare the input sequences with these stored mental representations, which, however, to the best of our knowledge have not been examined by previous studies.

In addition to sampling variability and pitch-processing complexity, inherent shortcomings in the methods used in previous behavioural studies could also contribute to the mixed findings regarding pitch processing in ASD. First, the dependent variable most often considered in these studies is the performance score for the task (e.g. percentage of correct answers), which does not allow us to rule out differences in processing strategies; for instance, autistic and non-autistic individuals might exhibit similar behavioural performance in discriminating sounds with a rising versus falling tone but still make use of different cues. As such, looking at performance scores alone might be misleading about the underlying processing differences between individuals, since a lower performance score can equally point towards a degraded pitch processing or to an increased level of internal noise, that is, the intrinsic variability in participants’ responses to the same stimuli. Regarding the latter aspect, several studies observed that autistic individuals exhibit higher levels of internal noise for processing sensory information compared to non-autistic individuals and that the level of internal noise uncovered correlates with individuals’ autistic traits (Park et al., 2017; Vilidaite et al., 2017). To fully understand the underlying mechanisms in pitch processing between autistic and non-autistic individuals, it is thus critical to opt for measurement methods providing a richer picture of pitch processing, including internal noise, than those obtained from performance scores alone. Second, in previous studies, the signal features (e.g. pitch) that drive judgements are limited regarding diversity across individuals and are chosen based on previous works (i.e. in a hypothesis-driven manner), which are very likely to create a variety of confirmation biases (Burred et al., 2019). For instance, prosodic cues in speech stimuli are normally generated by one or a few individuals (speakers) and then selected/confirmed by experimenters, but these cues may not exhaust the many other ways in which individuals express prosody and may not match individuals’ internal representations. Finally, the stimuli used in these studies generally stem from a limited number of utterances or corpora (e.g. from 20 to 100 stimuli), which may not be sufficient to capture sensitivity to subtle signal changes due to the likelihood of producing coarse variation (Ponsot, Burred, et al., 2018). It could be the case that autistic individuals have, in general, an impairment to process complex, unexpected temporally varying signals, but that they have acquired a better ability to process one specifically varying feature of the sound. Testing this with a restricted number of stimulus conditions, or in a task that does not require a direct top–down comparison of the incoming stimulus with their internal template might obscure the differences between autistic and non-autistic individuals or at worst, bias the results in one direction.

Taken together, this study explored a novel data-driven method, called the reverse-correlation paradigm, which allows us to directly and specifically focus on how top–down representations are used to process incoming stimulus (Brinkman et al., 2017; Gosselin & Schyns, 2002; Imhoff et al., 2013; Ratner et al., 2014) and has the potential to overcome the shortcomings related to previous approaches identified above. Specifically, in this paradigm, participants’ mental representations driving judgements can be mathematically determined and visually exposed by analysing the behavioural responses to large sets of systematically varied stimuli, without any restriction regarding the number and the space of varying features (Burred et al., 2019), and the level of internal noise can be directly estimated for each individual by assessing how well their mental representations can account for trial-by-trial responses associated to each pair of these randomly manipulated stimuli (Neri & Levi, 2006). Thus, by using reverse correlation, it allows portraying the pitch-processing characteristics of each individual much more precisely than through scalar metrics (e.g. performance and sensitivity). In addition, by deploying the exact same experimental design on speech and musical signals (see below), it offers a common ground to compare the mental representations of pitch between speech and music in both autistic and non-autistic individuals, and as such has the potential to inform the theoretical debate on whether speech and music share underlying mechanisms.

To that end, this study used a reverse-correlation approach based on speech, complex tone, as well as melodic stimuli that contain pitch structure, to assess and compare mental representations of pitch contours in each domain for autistic and non-autistic individuals. While for the melody condition, the consistency between participants’ representation of melodic contour and the theoretical musical notation could be directly estimated, there was no objective criterion to assess the ‘correctness’ of mental representations in the speech and complex tone conditions (e.g. there is no universal definition of what constitutes a ‘correct’ rising tone). We could, nonetheless, compare whether the groups differed in their representations across speech and complex tone conditions, and explore whether the differences between groups were driven by dissociable pitch-processing ability across conditions. Thus, based on previous findings suggesting that autistic individuals show atypical speech pitch perception and enhanced musical pitch perception relative to non-autistic individuals, we hypothesized that there would be subtle group differences in how they represent pitch in both speech and complex tone conditions, whereas in the melody condition, autistic participants would show a more consistent mental representation of the musical notation of the melody relative to non-autistic individuals.

## Methods

### Participants

Following the age cut-offs for the Autism-Spectrum Quotient (Baron-Cohen et al., 2001), we only recruited children (4–11 years) and adolescents (12–15 years) for the study. Thirty-two autistic individuals (5 females and 27 males) and thirty-two non-autistic individuals (5 females and 27 males), aged between 7.00 and 15.69 years, participated in the experiment. All participants were native speakers of Mandarin, and all non-autistic participants had no history of any neurological or psychiatric disorders, according to parent reports. All the autistic individuals had a clinical diagnosis of ASD, which was further confirmed using the Autism Diagnostic Observation Schedule – Second Edition (ADOS-2; Lord et al., 2012) by the first author (with clinical and research reliability for administration and scoring). All participants had normal hearing in both ears, with pure-tone air conduction thresholds of 25 dB HL or better at frequencies of 0.5, 1, 2 and 4 kHz. The participants completed a non-verbal intelligence quotient (IQ) test using the Raven’s Standard Progressive Matrices Test (RSPM) (Raven et al., 1998) and a receptive vocabulary test using the Peabody Picture Vocabulary Test-Revised (PPVT-R; Dunn & Dunn, 1981). The Chinese version of the Digit Span task (Wechsler, 2003) was used to assess verbal short-term memory. Table 1 shows the characteristics of the participants, as well as the results of Welch two-sample *t*-tests comparing group performance. The standardized scores for RSPM were calculated based on the means and standard deviations obtained from a Chinese normative study (Zhang, 1989), and only those participants with an IQ in the normal range (> 70) were included. Given that the Chinese norms for PPVT-R only included ages from 3.5 to 9 (Sang & Miao, 1990), standardized scores were calculated based on American norms (Dunn & Dunn, 1981). Correlation analysis revealed a significant positive relationship between the standardized scores obtained based on the Chinese norms and those based on the American norms (*r* = 0.95) for participants at or below 9 years old, thus confirming the validity of this approach. As can be seen, the two groups were matched in terms of age, years of musical training received, non-verbal IQ, and verbal short-term memory, but the ASD group manifested lower verbal ability compared to the non-ASD group. Written informed assent/consent was obtained from participants and their parents prior to the experiment, with the experimental procedures approved by the University of Reading Research Ethics Committee. There was no community involvement in the reported study.

**Table 1.** Characteristics of the ASD (*n* = 32) and non-ASD groups (*n* = 32).

Variables	ASD	Non-ASD	*t*	*p*	Cohen’s *d*
Age					
Mean (SD)	10.52 (2.42)	11.47 (2.75)	−1.47	0.15	−0.37
Musical training					
Mean (SD)	1.00 (1.37)	0.47 (1.78)	1.72	0.09	0.43
RSPM					
Mean (SD)	111.07 (14.88)	112.94 (9.98)	−0.59	0.56	−0.15
PPVT-R					
Mean (SD)	126.81 (25.90)	141.41 (12.83)	−2.86	**0.006**	−0.71
Digit span					
Mean (SD)	8.47 (1.08)	8.13 (1.10)	1.26	0.21	0.32

ASD: autism spectrum disorder; Musical training: years of musical training; RSPM: standard score of Raven’s Standard Progressive Matrices Test; PPVT-R: standard score of Peabody Picture Vocabulary Test-Revised; Digit span: raw score of verbal short-term memory.

### Stimuli

There were three types of auditory stimuli: speech, complex tone and melody. For the speech stimuli, a single word 眯 /mi/ (‘to squint’) with a high tone in Mandarin was recorded by a native female adult speaker. The original sound was manipulated to last 250 ms, with the intensity set at 80 dB and original pitch contour flattened to its mean pitch (210 Hz) using Praat (Boersma & Weenink, 2001). Following a previous study, a Python-based toolbox (CLEESE; see Burred et al. (2019) for details) was used to generate variations of the sound with randomly manipulated pitch contours while maintaining a constant amplitude and duration. Specifically, Gaussian pitch noise (i.e. pitch-shifting) was added to the contour by sampling pitch values at eight successive time points, using a normal distribution (SD = 70 cents; clipped at ±2.2 SD). The values at the eight successive time points were linearly interpolated between time points and were used to compute participants’ mental representations of pitch contours. After piloting (see the piloting section below), a total of 800 speech stimuli were synthesized.

For the complex tone stimuli, Praat was used to generate a complex tone analogue of /mi/. The complex tone comprised of F0 (fundamental frequency) and its seven odd harmonics, of the same amplitude and with sine phase, which leads to a clarinet sound quality (Liu et al., 2010; Patel et al.,1998, 2005, 2008). In keeping with the acoustic characteristics of the speech sound /mi/, the pitch value of the complex tone was set at 210 Hz, intensity at 80 dB, and duration at 250 ms. Thereafter the same procedure was applied as with the speech stimuli to manipulate the pitch contour of the complex tone across eight time points. Based on findings from previous studies (Burred et al., 2019; Ponsot, Burred, et al., 2018) and on how pitch targets are realized during speech production (Xu & Wang, 2001), we hypothesized that, among the eight time points in the speech and complex tone conditions, later time points would carry more weights than the earlier time points in driving participants’ judgements of the rising pitch. After piloting (see the piloting section below), 800 complex tones with different pitch contours were generated.

**Figure 3.**
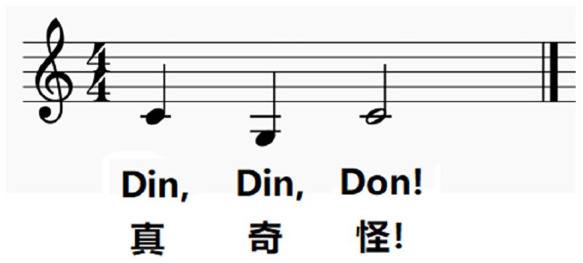
The musical notation of the last phrase of ‘Frère Jacques’ and ‘Two tigers’.

For the melody, the last phrase of a popular Chinese nursery rhyme ‘Two Tigers’, ‘真奇怪’ (‘It’s so weird!’), was recorded by a Mandarin-speaking female singer. The song has the same tune as the French nursery rhyme ‘Frère Jacques’ (See Figure 3). The onsets of the three sung notes in the phrase were manually identified and used as breakpoints. The pitch contour was then artificially flattened to its mean pitch (260 Hz). Next, we used CLEESE to add Gaussian pitch noise to the contour by sampling pitch values at the three time points (i.e. the onset of each sung note), using a normal distribution (SD = 70 cents; clipped at ±2.2 SD) and a square breakpoint function (BPF) with a transition time of 0.1 s. We hypothesized that all three time points would contribute to participants’ judgements of the melodic contour. The duration of the stimulus was kept at 1380 ms. After piloting (see the piloting section below), 600 non-identical versions of this phrase were generated using CLEESE.

### Piloting

Reverse-correlation experiments typically have many trials to obtain reliable results (Ponsot, Arias, et al. (2018) had 500 trials and Park et al. (2017) used 480 trials). Given that each participant would have to complete three different conditions (speech, complex tone and melody) to obtain reliable results while keeping the duration of each condition feasible, a pilot study was conducted to determine the optimal number of trials for each condition. Burred et al. (2019) showed that reliable results (defined as *r* = 0.8 and above for the correlation of pitch values between a subset of trials and the full set of trials) were obtained by most participants within 100 trials, though some needed up to 300 trials. Based on that, we conducted a pilot study with 1000 stimuli in speech and complex tone conditions. Since the melodic phrase was relatively long, we included 600 stimuli for the melody condition. Stimuli were randomly paired in each condition and three Mandarin-speaking children with typical development made judgements on each pair per the requirement of the condition (e.g. in the speech condition, participants judged which of the pair was a /mi/, ‘迷’ (‘confused’ or ‘lost’), with a rising pitch). Using a similar approach to Burred et al. (2019), preliminary data from these three participants showed that reliable results were obtained with approximately 300 trials in the speech and complex tone conditions and 150 trials in the melody condition (see Supplementary information (SI) Appendix Figure S1). Given these findings, it was determined that 400 trials in the speech and complex tone conditions and 300 trials for the melody condition would be appropriate for the main experiment.

### Procedure

As in the pilot, the main experiment consisted of three separate conditions – speech, complex tone and melody. To avoid potential fatigue or boredom of the participants, the three conditions were administered during different visits when participants also took part in other experiments. The presentation order of the three conditions was counterbalanced across participants, with no constraint on the number of days in between the visits, which depended entirely on participants’ availability. In the speech condition, participants listened to 400 pairs of randomly modulated /mi/ and were asked to indicate which of the two in each pair best matched the meaning of /mi/ with a rising tone, represented with the Chinese character ‘迷’ (‘confused’ or ‘lost’). In the complex tone condition, participants heard 400 pairs of complex tones and chose the one that best matched a rising tone, indicated by an arrow on the screen (↗). In the melody condition, 300 pairs of randomly manipulated sung phrases were presented to the participants, who had to judge which of each pair best resembled the melody of the last phrase in ‘Two Tigers’. The stimulus pairings in each condition were randomized across participants, with an inter-stimulus interval of 500 ms and an inter-trial interval of 1 s across all three conditions. Participants were given the option to take a self-timed break after every 100 trials in each condition, which corresponded roughly to after every 7 min for the speech/complex tone condition and after every 10 min for the melody condition. To maintain their attention, participants were required to make their responses orally for the experimenters to input into the computer. The entire experiment with all three conditions took about two h (with breaks) in total, with each condition lasting about 40 min.

### Data analysis

For each participant, we computed a first-order temporal kernel (Ahumada & Lovell, 1971) separately for speech, complex tone and melody conditions. For each condition, the kernel was calculated as the mean pitch contour difference between the stimuli that were chosen as the best match in each pair and those that were not chosen.

Using these kernels, group differences in participants’ mental representations of the pitch contours were compared across the three conditions in several different ways. First, speech and complex tone data were analysed using a linear mixed-effects model to compare group differences at each time point of the temporal kernel. The linear mixed-effects model was performed using the *lme4* (Bates et al., 2012; Brauer & Curtin, 2018) and *lmerTest* (Kuznetsova et al., 2017) packages with pitch value at each time point as the dependent variable and group (ASD vs non-ASD), stimuli type (speech vs complex tone), time point (T1–T8) as well as all possible interactions as fixed effects. For random effects, the model was fitted with by-subject intercepts, and the random slopes of stimuli type and time point were initially included but were later removed due to convergence issues. Second, to further detect any potential differences in overall shape of the kernel between groups for each condition, linear and nonlinear models (quadratic, cubic and quartic) were fit to the kernels of each group and condition. The models were compared using likelihood-ratio tests to determine which model/shape provided the best fit for the data. This was done to determine whether the groups differed in the overall shape of their temporal kernels for each condition. For example, for the speech condition, if a linear model fitted the data of the ASD group best whereas a cubic model fitted the non-ASD data best, then this would suggest a group difference in the overall shape of the kernels, which might potentially not have been revealed in the linear mixed-effects model. Finally, after determining the best model for each group and condition, the appropriate model was fit for each participant by group and condition and two individual-level parameters of the model – *y*-intercept and slope – were calculated. Group differences in these parameters were then compared using two-sample *t*-tests. For the melody data, similarly, we first used a linear mixed-effects model to compare group differences at each note/time point of the temporal kernel. Specifically, pitch values at each note were added as the dependent variable, and note (N1–N3) and group were included as the fixed effects, with by-subject intercepts as the random effects. We also used correlations to compare the matching of pitch contours to the musical notation of the melody for each group separately.

To assess the energy of the kernels obtained, two further parameters were computed by participants and conditions. First, the root-mean-square (RMS) value was calculated for each participant’s kernel under each condition, which is a scalar of pitch perceptual filter that reflects how much individuals weight the different pitch portions in one direction or the other (Ponsot et al., 2020). The higher the RMS, the more sensitive the participant is to the pitch. Groups were then compared on their RMS values for each condition using two-sample *t*-tests. Second, internal noise, which reflects non-systematic variations in participants’ perceptual responses, was measured to rule out the possibility that any group difference in their mental representations was due to random variations in their responses (Burred et al., 2019; Venezia et al., 2019). For each participant, it was first determined which of the pair on each trial was the most similar to the individual’s kernel (i.e. the individual’s mental representation) for each condition using correlation analysis (see the study by Neri & Levi, 2006). The stimulus with the higher correlation coefficient in each pair was defined as the objectively correct response on each trial. Next, the percentage of the participant’s actual responses that agreed with those of the objectively correct responses using correlation analysis was computed, with the assumption that this percentage would be an index of internal noise (higher consistency values reflect lower internal noise). Groups were then compared on their internal noise for each condition using two-sample *t*-tests. These two parameters were used to obtain how sensitive individuals were to pitch while also estimating the extent to which random responses contribute to representation. All statistical analyses were conducted using RStudio (RStudio Team, 2020). Subsequent post hoc comparisons, if any, were conducted using the *emmeans* package (Lenth et al., 2018).

## Results

### Age, gender and receptive verbal ability considerations

In this study, aside from the differences in PPVT-R scores, the two groups were comparable in other cognitive and demographic variables, including non-verbal IQ, verbal short-term memory, gender, age and years of musical training. However, the age range was relatively large (7.00–15.69 years) and the gender ratio was unbalanced between male and female participants (5 F:27 M). Hence, we considered age and gender along with the PPVT-R as fixed effects in preliminary statistical analyses to examine the potential effects of these variables. Since none of these factors were likely to be a contributing factor to any of the differences we report (all *p*-values > 0.05), these factors were subsequently removed from the models (see SI Appendix Table S1).

### Speech versus complex tone

#### Comparison at each time point

**Figure 4.**
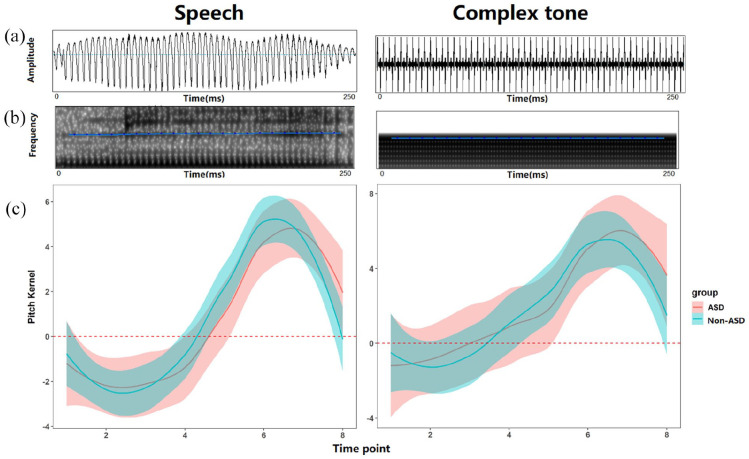
The (a) waveform, (b) spectrogram and level contour (blue line) of the original sound and the (c) pitch kernels of the rising pitch for speech and complex tone among ASD and non-ASD groups (shaded areas show 95% confidence interval).

Figure 4 displays the pitch kernels of speech and complex tone for each group. The linear mixed-effects model revealed significant main effects of time point (*F*(7,930) = 27.03, *p* < 0.001) and stimulus type (*F*(1,930) = 32.90, *p* < 0.001), as well as a significant interaction between the two (*F*(7,930) = 3.82, *p* < 0.001). Post hoc analyses with Bonferroni correction indicated that the pitch values in the speech condition were significantly lower than those in the complex tone condition at time point 3 (speech: M = −4.38, SD = 7.13 vs complex tone: M = −0.42, SD = 6.55; *t*(931) = 3.30, *p* = 0.01), time point 4 (speech: M = −3.04, SD = 6.29 vs complex tone: M = 1.54, SD = 4.89; *t*(931) = 3.82, *p* < 0.001), and time point 7 (speech: M = 2.18, SD = 6.58 vs complex tone: M = 7.8, SD = 14.34; *t*(931) = 4.69, *p* < 0.001), but not at other time points. In addition, no significant main effects of group or group-related interactions were observed, suggesting that the two groups showed comparable pitch kernels across speech and complex tone conditions along the eight time points.

### Comparison of the overall shape

Linear and nonlinear polynomial models (quadratic, cubic and quartic) were fitted to each group and condition and the best-fitting model was selected using likelihood-ratio tests, and the results are shown in Table 2 (see SI Appendix Results S1 for further details about model comparisons).

**Table 2.** Results of likelihood-ratio tests to determine the best-fitting model by group and condition.

Condition	Group	Comparison	DF	F	*P*	The better fitting model	The best-fitting model
Speech	ASD	Linear and Quadratic	1	0.71	0.40	Linear	Cubic model
		**Linear and Cubic**	**2**	**10.48**	**<0.001** ***	**Cubic**	
		Cubic and Quartic	1	1.18	0.28	Cubic	
	Non-ASD	Linear and Quadratic	1	1.26	0.26	Linear	Cubic model
		**Linear and Cubic**	**2**	**26.00**	**<0.001** ***	**Cubic**	
		Cubic and Quartic	1	0.71	0.40	Cubic	
Complex tone	ASD	Linear and Quadratic	1	0.15	0.70	Linear	Quartic model
		**Linear and Cubic**	2	2.68	0.07	Linear	
		Linear and Quartic	**3**	**3.07**	**0.03** *	**Quartic**	
	Non-ASD	Linear and Quadratic	1	2.20	0.14	Linear	Cubic model
		**Linear and Cubic**	**2**	**10.43**	**<0.001** ***	**Cubic**	
		Cubic and Quartic	1	2.30	0.13	Cubic	

ASD: autism spectrum disorder.

* *p* < 0.05; **p < 0.01; ***p < 0.001.

After determining the best models to fit the data by condition and group, we computed the *R*^2^ for each model to ensure that the groups did not differ significantly in the proportion of the variance explained by the model fit for each condition for a fair comparison in their mental representation. For the speech condition, a two-sample *t*-test showed no significant difference between the ASD and non-ASD groups in their *R*^2^ values (*t*(61.75) = 0.14, *p* = 0.89; ASD: M = 0.54, SD = 0.29; non-ASD: M = 0.53, SD = 0.27). Similarly, for the complex tone condition, the two groups did not differ significantly in *R*^2^ (*t*(60.83) = 1.95, *p* = 0.06; ASD: M = 0.62, SD = 0.23; non-ASD: M = 0.50, SD = 0.26). Given so, we found that a cubic model was the best-fitting model for both groups in most cases, except for the complex tone data, where the quartic shape was selected as the best-fitting model for the ASD group.

To compare whether there were any subtle differences in magnitude or slope of the overall shapes between groups for each condition, we extracted two model parameters – *y*-intercept (b0) and slope of tangent at midpoint – from the models fitted to each participant. Two-sample *t*-tests revealed no significant group difference in either *y*-intercept (speech: *t*(53.17) = 0.62, *p* = 0.54 vs complex tone: *t*(50.44) = 0.38, *p* = 0.70) or slope (speech: *t*(58.59) = 0.72, *p* = 0.47 vs complex tone: *t*(32.18) = 1.64, *p* = 0.11) in both conditions.

In short, these results indicate that the two groups exhibited similar morphology (cubic shape) for the speech kernels, while displaying different overall shapes for the complex tone kernels (quartic in ASD vs cubic in non-ASD).

### Melody task

**Figure 5.**
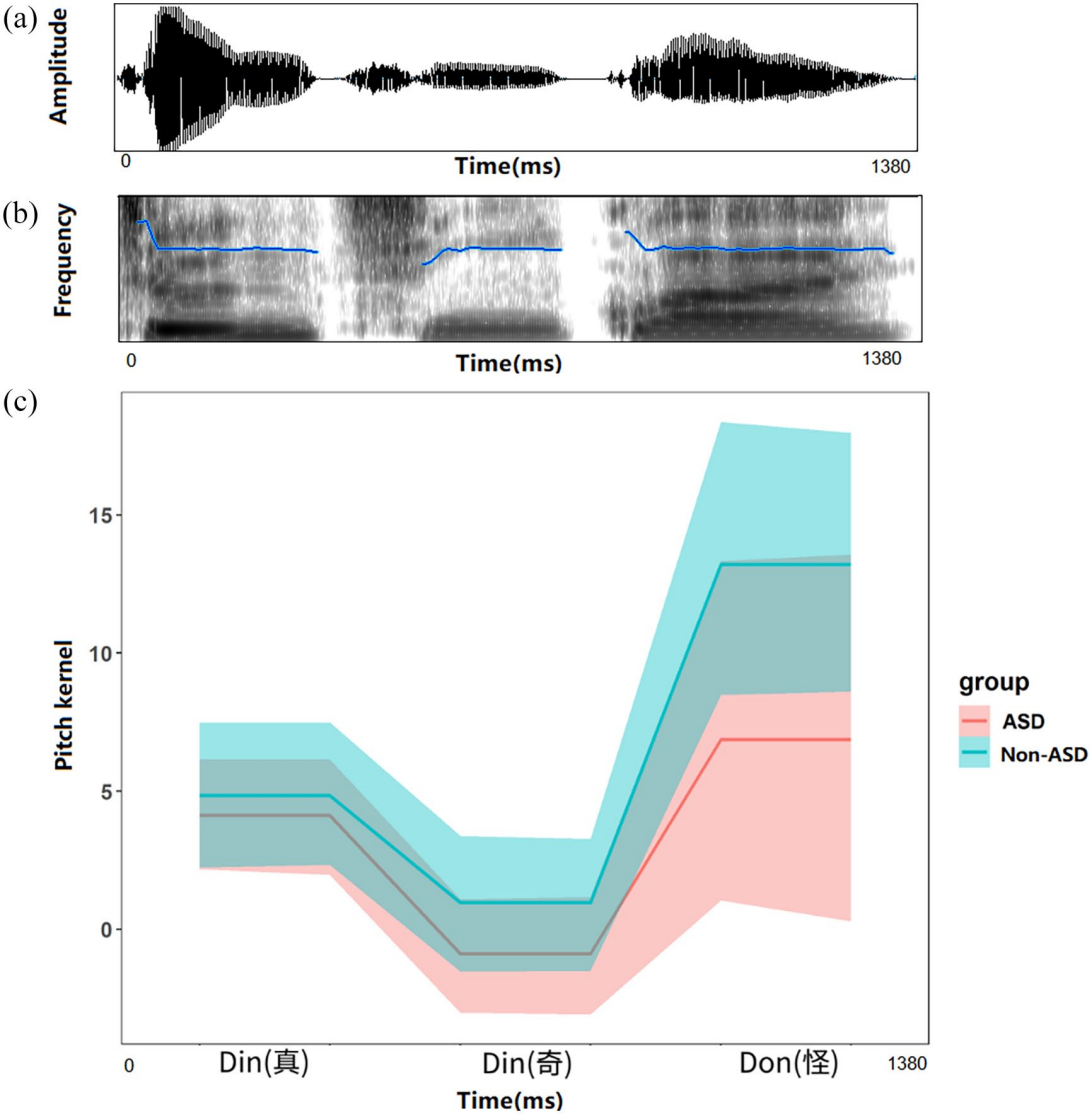
The (a) waveform, (b) spectrogram and level contour (blue line) of the original phrase and the (c) pitch kernels of the melody in ASD and non-ASD groups (shaded areas show 95% confidence interval).

Figure 5 displays the pitch kernels of the melodic phrase for each group. The linear mixed-effects model revealed a significant main effect of note (*F*(2, 124) = 15.61, *p* < 0.001). Post hoc analyses with Bonferroni correction revealed that the pitch values at Note 2 were significantly lower than those at Note 1 (Note 1: M = 4.48, SD = 6.85 vs Note 2: M = 0.04, SD = 6.54; *t*(124) = 2.48, *p* = 0.04) and at Note 3 (Note 3: M = 10.04, SD = 16.59; *t*(124) = 3.10, *p* = 0.007). Also, Note 1 was significantly lower than Note 3 (*t*(124) = 5.58, *p* < 0.01). The main effect of group and interaction between group and time point did not reach significance (all *p*-values > 0.05), suggesting that the two groups showed a similar mental representation of the melody.

We computed Pearson correlations between the mean kernel of each group and the prototypical melodic contour of the phrase based on musical notation (i.e. the second note is five semitones lower than the first, and the third note is five semitones above the second; see Figure 3). Results showed that the kernels from both groups were positively correlated with the prototypical melodic contour (ASD: *r*(1) = 0.94, *p* = 0.23; non-ASD: *r*(1) = 0.74, *p* = 0.47), with the correlation coefficient of the ASD group being higher than that of the non-ASD group. We then calculated the correlation for each individual and compared the coefficients between ASD and non-ASD groups. Results suggested that participants from the two groups did not differ significantly in their correlation coefficients (*t*(61.46) = 0.10, *p* = 0.92; ASD: M = 0.43, SD = 0.51; non-ASD: M = 0.42, SD = 0.56). In summary, both groups showed a mean pitch kernel that was consistent with the prototypical melodic contour, with the ASD group having a higher (albeit non-significant) correlation coefficient than the non-ASD group.

### RMS values and internal noise

**Figure 6.**
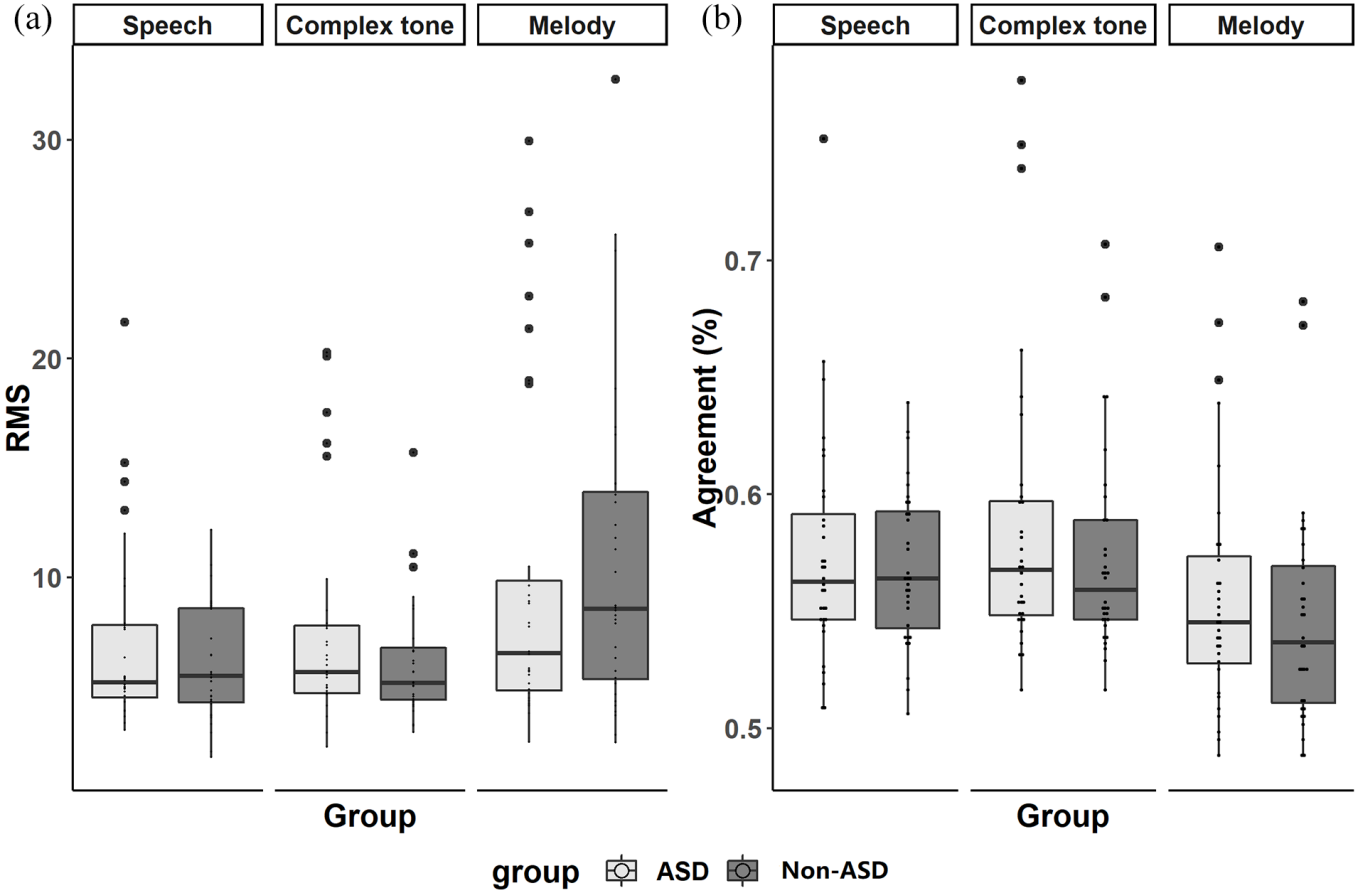
The results of (a) RMS values of perceptual filters and (b) agreement percentage, an index of internal noise in both groups.

Figure 6 displays the results of RMS values (as measured using the kernel of each participant by condition) and the internal noise (as measured using the percentage of agreement between the objectively correct response as dictated by the participant’s kernel and their actual response on each trial) by each group and condition. Two-sample *t*-tests showed that the RMS values were not significantly different between the groups in any of the conditions, and nor was the internal noise (all *p*-values > 0.05, see Table 3). RMS values were significantly positively correlated with the agreement percentage in both groups and under both conditions (all *p*-values < .001, see SI Appendix Figure S2), which is in line with theoretical expectations (Murray, 2011; Ponsot et al., 2020) indicating that the more inter-trial stochastic variability, the lesser energy in the estimated kernels. In addition, measures of RMS and internal noise were independent of ASD severity levels for autistic participants (see SI Appendix Results S2 for details).

**Table 3.** The results of two-sample *t*-tests on RMS values and internal noise (agreement).

		Mean (ASD: non-ASD)	SD (ASD: non-ASD)	*t*	df	*p*
Speech	RMS	7.00:5.99	4.20:2.58	1.16	51.49	0.25
	Agreement	0.57:0.57	0.05:0.03	0.49	54.09	0.62
Complex tone	RMS	7.56:6.32	4.86:3.17	1.20	53.35	0.23
	Agreement	0.59:0.57	0.06:0.04	1.02	55.05	0.31
Melody	RMS	9.92:10.7	7.7:7.17	0.42	61.69	0.68
	Agreement	0.56:0.55	0.05:0.05	0.89	61.31	0.38

ASD: autism spectrum disorder; RMS: root mean square.

### Subgroup analysis

Given previous reports indicating that enhanced pitch perception may be a characteristic of a small autistic subgroup (Heaton, Williams, et al., 2008; Jones et al., 2009; Kargas et al., 2015), we further explored the participants’ RMS in each task to determine whether there was a subgroup of autistic individuals with exceptional pitch sensitivity in this study. Exceptional pitch sensitivity in each task was defined as an RMS value two SDs above the mean RMS value of the non-ASD group. For the speech and complex tone tasks, five autistic participants (15.63% of the ASD group) showed exceptional pitch sensitivity above two SDs of the non-ASD group mean (speech: M = 11.16, *SD* = 2.58; complex tone: M = 12.67, *SD* = 3.17), compared to one (3.13% of the non-ASD group) in the speech task, and two non-autistic participants (6.25% of the non-ASD group) in the complex tone task. In addition, the proportion was similar between groups for the melody task (M = 25.04, *SD* = 7.17), with three autistic participants (9.38%) and two non-autistic participants (6.25%) performing two SDs above the non-ASD group mean (see SI Appendix Table S2 for the characteristics of participants who showed exceptional pitch sensitivity).

### Intra-group variability

To examine the amount of intra-group variability in different conditions regarding kernel shapes, we computed the correlation coefficient between participants by group. Then, we compared the distributions of the correlation coefficients between groups for each condition using two-sample *t*-tests (Table 4). Results showed that the between-participants correlations in the ASD group were significantly lower than those in the non-ASD group in speech and melody conditions, suggesting that the intra-group variability was higher in ASD.

**Table 4.** The results of two-sample *t*-tests on intra-group variability.

		Mean	SD	*t*	df	*p*
Speech	ASD	0.11	0.41	5.20	988	< 0.001
	Non-ASD	0.24	0.39			
Complex tone	ASD	0.06	0.42	1.72	988	0.08
	Non-ASD	0.10	0.40			
Melody	ASD	0.13	0.71	3.35	988	< 0.001
	Non-ASD	0.28	0.69			

ASD: autism spectrum disorder.

## Discussion

This work capitalized on a reverse-correlation paradigm to characterize the mental representation of pitch contours in Mandarin-speaking autistic and non-autistic individuals and across different auditory domains (speech vs complex tone vs melody), a component of pitch processing that maximally engages top–down representations but has remained overlooked in previous studies. Groups were compared in terms of their representation of speech and musical pitch contours in two ways: (1) by examining group differences at each time point and (2) by examining the global shape of their representations. The two groups did not differ significantly at any time point in their mental representations of pitch contours across speech, complex tone and melody conditions. When considering the global shape of mental representations, while autistic and non-autistic participants showed a different overall shape in the representation of the complex tone pitch contour (quartic shape for the ASD group and cubic shape for the non-ASD group), the two groups exhibited a similar cubic shape in their representation of the rising pitch contour in speech. Concerning the melody condition, the two groups manifested similar global patterns overall, which positively correlated with the prototypical melodic contour. In addition, the two groups did not differ significantly in RMS values (pitch perceptual filter) across conditions, providing evidence for similar pitch sensitivity between the two groups across domains. However, a subgroup of autistic individuals did show exceptional pitch sensitivity, supporting the notion that enhanced pitch processing might only be present in some autistic individuals. Furthermore, the intra-group variability was significantly higher in the ASD group than in the non-ASD group. Given that the two groups did not differ significantly in measures of their internal noise, these results are likely to reflect their genuine ability in representing pitch, rather than due to random variations in their responses. Taken together, the present findings suggest for the first time that, while autistic participants exhibit diverse profiles of pitch processing compared to non-autistic individuals, their mental representations of pitch contours show similar patterns to non-autistic individuals across speech and music domains.

Using a comparative design, the present findings indicate that top–down processing of pitch may constitute domain-general mechanisms across speech and music in ASD. These findings provide theoretical implications for using music therapy or song-based interventions to help improve language and communication in ASD, especially for those with communication deficits (Geretsegger et al., 2014; Sharda et al., 2015). Indeed, previous neuroimaging studies suggest that the neural systems subserving song are preserved in ASD, whereas those for speech show reduced patterns compared to non-autistic individuals (Lai et al., 2012; Sharda et al., 2015). Also, a recent systematic review investigated the benefits of music in rehabilitation of communication disorders and revealed that in addition to improving joint attention and group participation, music-based therapy enhanced social communication skills, including an increased frequency of verbal and non-verbal responses, initiating vocalizations and taking conversational turns (Boster et al., 2021). However, more research is needed to explore whether and how song-based interventions could improve language production and comprehension in ASD (Williams et al., 2021).

Consistent with our hypothesis regarding complex tone pitch processing, the two groups exhibited different overall shapes of mental representations of a rising pitch contour. However, it was unlikely that this difference was driven by enhanced or impaired complex tone pitch-processing ability in autistic participants as suggested by previous studies (Bonnel et al., 2003, 2010; Heaton et al., 1998; Kargas et al., 2015; O’Riordan & Passetti, 2006), for several reasons.First, as indicated by RMS values, the two groups had similar perceptual sensitivity to pitch in the complex tone condition. Second, the two groups did not differ significantly at any time point of the complex tone pitch contours. Finally, both groups showed a final increase in pitch at the end of the complex tone (see Figure 4). Taken together, we argue that the differences between groups are more about variations in how they represent rising tones, rather than being driven by enhanced or impaired musical pitch processing in the ASD group.

A comparable mental representation of pitch contour in the speech condition between the ASD and non-ASD groups is compatible with previous findings suggesting that autistic individuals may have no impairment in speech pitch processing (Cheng et al., 2017; Wang, Beaman, et al., 2021). However, this stands in contradiction to other findings of either impaired (Jiang et al., 2015; Paul et al., 2005) or enhanced speech pitch perception in autistic individuals (Heaton, Hudry, et al., 2008; Järvinen-Pasley et al., 2008; Järvinen-Pasley & Heaton, 2007). These discrepancies may be explained by several possible reasons, the first being the fundamental differences in methodologies used among these studies. In particular, as mentioned in the ‘Introduction’ section, previous studies used standard behavioural tasks (e.g. discrimination and identification) to investigate whether autistic individuals can perceive pitch differences in speech based on their response accuracy (Heaton, Hudry, et al., 2008; Järvinen-Pasley et al., 2008; Järvinen-Pasley & Heaton, 2007; Jiang et al., 2015; Paul et al., 2005; Wang, Beaman, et al., 2021). In those tasks, the higher the response accuracy, the better the pitch-processing ability. However, these measures do not reveal the potential differences in processing strategies that participants may employ, for example, how participants compare internal mental representations of time-varying pitch contours with incoming sounds. This study used a reverse-correlation paradigm to explore whether autistic individuals manifest an impaired top–down processing ability to compare the input sequences with stored mental representations for speech sounds. Thus, this study and previous studies were designed to address two different research questions, mental pitch representation versus auditory pitch perception. Studies that used the same paradigm within the visual domain suggested a top–down effect between representation and perception (Brinkman et al., 2017). We, therefore, speculated that the fidelity of how well one represents pitch might be associated with behavioural judgements of pitch. For example, a better pitch representation might relate to a more accurate judgement of pitch as measured by standard behavioural tasks. Nevertheless, given that this study did not test behavioural outcomes of pitch processing (e.g. pitch identification/discrimination accuracy, etc.), this speculation needs to be investigated by future studies applying both the reverse-correlation paradigm and standard behavioural measures on the same participants. Another reason may be due to sampling variability across studies. Indeed, we found greater intra-group pitch variability in the ASD group compared with the non-ASD group. This finding supports studies claiming that heterogeneity is a prominent feature of ASD (Kargas et al., 2015; Milne, 2011; Valla & Belmonte, 2013). However, the present findings of intact mental representations of speech pitch contours in ASD may not be generalized to a less cognitively capable ASD group, who may not be able to perform the experiments due to task complexity and cognitive demands. Further studies employing alternative methods are required to replicate the current findings across the autism spectrum and from people with different language backgrounds.

Regarding melody, the finding of intact/enhanced melodic pitch perception is consistent with the main findings from previous musical studies (see the study by Heaton, 2009 for a review). Autistic and non-autistic participants showed a comparable melodic pitch contour, suggesting that they represent the pitch contour of the well-known melody in a similar manner. When comparing the consistency with the prototypical melodic contour, the ASD group showed a more consistent representation of the melody than the non-ASD group. This suggests a superior ability to perceive and assess scaled versions of the target musical melody in the ASD group, although the difference in consistency between groups did not reach significance. However, it is worth mentioning that the last note of the melody is longer than the first two (see Figure 5). Thus, it is possible that this long last note received a greater attention than the first two notes from the non-ASD group, resulting in a bias (i.e. upweight) in their pitch judgement on that note, whereas the ASD group mainly focused on the pitch dimension, ensuring that their pitch kernel was closer to the ground truth. This finding indicates that when the representation of musical pitch structure requires higher precision, for example, matching the prototypical melody, some autistic individuals may outperform non-autistic individuals.

The comparable internal noise between groups is inconsistent with the findings in the visual domain, where a higher internal noise has been suggested in the ASD group than the non-ASD group (Park et al., 2017). In addition to investigating different domains (auditory vs visual) between the present and those visual studies, the genuine ability of autistic individuals to process different information might also explain the conflicting findings. Specifically, Park et al. (2017) examined visual orientation discrimination in the presence of varying levels of external noise, and they found that in addition to increased internal noise, autistic participants also performed worse in response to external noise relative to non-autistic participants. This means that in contrast to this study where autistic participants showed intact ability to process pitch across speech and music conditions, autistic participants were impaired in responding to external visual noise when making orientation discrimination decisions in the study by Park et al. (2017). Thus, it appears that when processing the information autistic individuals are good at, for example, in this study, they do not show higher internal noise than non-autistic individuals, whereas when processing the information autistic individuals struggle with, an increased level of internal noise might accompany the degraded abilities. This also explains why this study did not observe the correlation between ASD severity level and internal noise. However, given that no previous auditory studies have investigated the relationship between perceptual performance and internal noise in ASD, nor between ASD severity level and internal noise, further studies are needed to consolidate the current assumption that there is a causal relationship between task difficulty and internal noise levels in ASD.

In summary, we assessed how well autistic individuals are able to represent pitch using acoustically matched speech sounds and complex tones, as well as melodies. One of the primary interests in doing this was to resolve previously inconsistent findings from a different aspect of the pitch process; that is, top–down comparisons of internal mental representations of time-varying pitch contours with incoming sound signals. In addition, by using acoustically matched speech sounds and complex tones, we could determine whether autistic individuals perform differently across domains, so as to inform the theoretical debate about whether speech and music share the same underlying mechanisms. Our findings indicate that autistic participants showed similar mental representations of speech and musical pitch contours, suggesting an intact top–down processing of pitch in both the speech and music domains. However, the ASD group had a higher intra-group variability than the non-ASD group, which might be the main reason for previously inconsistent findings regarding pitch processing in ASD. In addition to similar mental representations between autistic and non-autistic participants in speech and music, the two groups did not differ significantly either in pitch sensitivity (i.e. suggested by RMS values) or in internal noise. Hence, these results support the view that pitch processing constitutes domain-general mechanisms in ASD (Wang, Pfordresher, et al., 2021), which provides theoretical implications for using music-based interventions to improve speech for autistic individuals with speech communication deficits (Eigsti et al., 2011).

## Limitation

A limitation of this study is that we only focused on a subgroup of the ASD population who had comparable cognitive and demographic variables to their non-autistic peers, including non-verbal IQ, verbal short-term memory, age and years of musical training, due to our task demands. Thus, the mental representation of pitch contours of a less cognitively capable ASD group remains to be explored. However, the present results are clear in indicating that the group differences in intra-group variability and the similarities in mental representations of pitch contours across speech and music reported here cannot be explained in terms of the differences in receptive verbal ability. In addition, the age range for the current sample is relatively wide across child and adolescent cohorts, although there was no age effect observed in the results of this study. It has been suggested that while there is a clear developmental improvement between children and adults across several pitch-related tasks, including pitch direction discrimination, statement–question intonation discrimination, identification and imitation tasks, the developmental changes between children and adolescents are not always significant and are dependent on task demand (Wang, Beaman, et al., 2021). Thus, while there is no age-related difference between children and adolescents regarding how they mentally represent pitch contours in speech and music, as indicated by this study, it would be intriguing to examine the developmental changes between children and adults in future studies.

## Conclusion

In this study, a novel reverse-correlation paradigm was used to investigate for the first time how ASD affects mental representations of pitch contours in speech syllable, complex tone and musical melody. Our findings revealed that the representations were similar between autistic and non-autistic individuals across different domains. However, a diverse profile of pitch processing in ASD has also been uncovered from a top–down pitch-processing perspective. This study extends our understanding of pitch processing in ASD and pitch representation in speech and music, suggesting that pitch processing constitutes domain-general mechanisms in ASD. In addition, it demonstrates a novel and promising method for investigating auditory processing in ASD. Future studies should compare mental representations of pitch in ASD from different language backgrounds and cognitive abilities to consolidate the current results.

## Supplemental Material

sj-docx-1-aut-10.1177_13623613221111207 – Supplemental material for Mental representations of speech and musical pitch contours reveal a diversity of profiles in autism spectrum disorder Supplemental material, sj-docx-1-aut-10.1177_13623613221111207 for Mental representations of speech and musical pitch contours reveal a diversity of profiles in autism spectrum disorder by Li Wang, Jia Hoong Ong, Emmanuel Ponsot, Qingqi Hou, Cunmei Jiang and Fang Liu in Autism
