# Supplementary material for: Mental representations of speech and musical pitch contours reveal a diversity of profiles in autism spectrum disorder
Source: Autism. 2022 Jul 18;27(3):629–46. doi: 10.1177/13623613221111207 (PMC10074762; doi:10.1177/13623613221111207)
Supplement: sj-docx-1-aut-10.1177_13623613221111207 – Supplemental material for Mental representations of speech and musical pitch contours reveal a diversity of profiles in autism spectrum disorder [file sj-docx-1-aut-10.1177_13623613221111207.docx]

## **Supplementary Information for:**

# **Mental representations of speech and musical pitch contours reveal a diversity of profiles in autism spectrum disorder**

### **Results**

#### S1 Comparison of the overall shape

Taking the ASD group’s speech condition as an example, we first compared a linear model with a quadratic model and found that the quadratic model did not lead to a significantly improved fit over the linear model (F(1) = 0.71, *p =* 0.40); so the quadratic model was rejected. Then we compared the linear model with a cubic model, and we found that the cubic model provided a significantly better fit to the data (F(2) = 10.48, *p* < .001), and so we rejected the linear model. Finally, a cubic and quartic model comparison was conducted and the fit of the quartic model was not significantly better than that of the cubic model (F(1) = 1.18, *p =* 0.28). Therefore, the cubic model was the best model to fit individual kernels for the ASD group in the speech condition. The procedure was repeated for all the other conditions in each group, and we found that a cubic model was the best-fitting model in most cases except the complex tone data for the ASD group (see Table 2).

#### S2 The relationship between ASD severity and performance on internal noise and RMS

To explore the relationship between ASD severity levels and performance on internal noise and RMS across conditions, we used the comparison scores of ADOS (all participants with ASD were administrated using the ADOS Module 3) and values of agreement percentage and RMS. Comparison scores on ADOS range from 1 to 10 (4-10 are indicative of ASD), with 10 representing the highest severity of autism-related symptoms (Duda et al., 2014; Gotham et al., 2009). Kendall’s tau correlations indicated that there was no correlation between ASD severity level and internal noise/RMS (see Figure S3).

Figure S1. Correlations between the contours derived using the n first trials and the kernels derived using all trials (n = 500/300) in speech, complex tone and melody tasks.


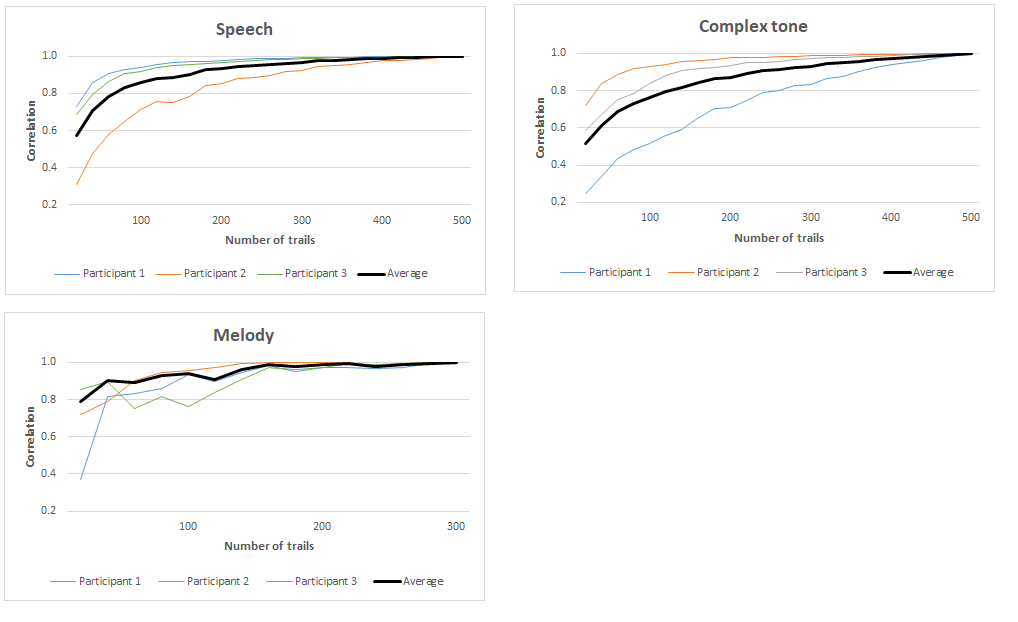


Figure S2. Relationship between RMS-values of the perceptual filters and the agreement percentage (Shaded areas show 95% Confidence Interval).


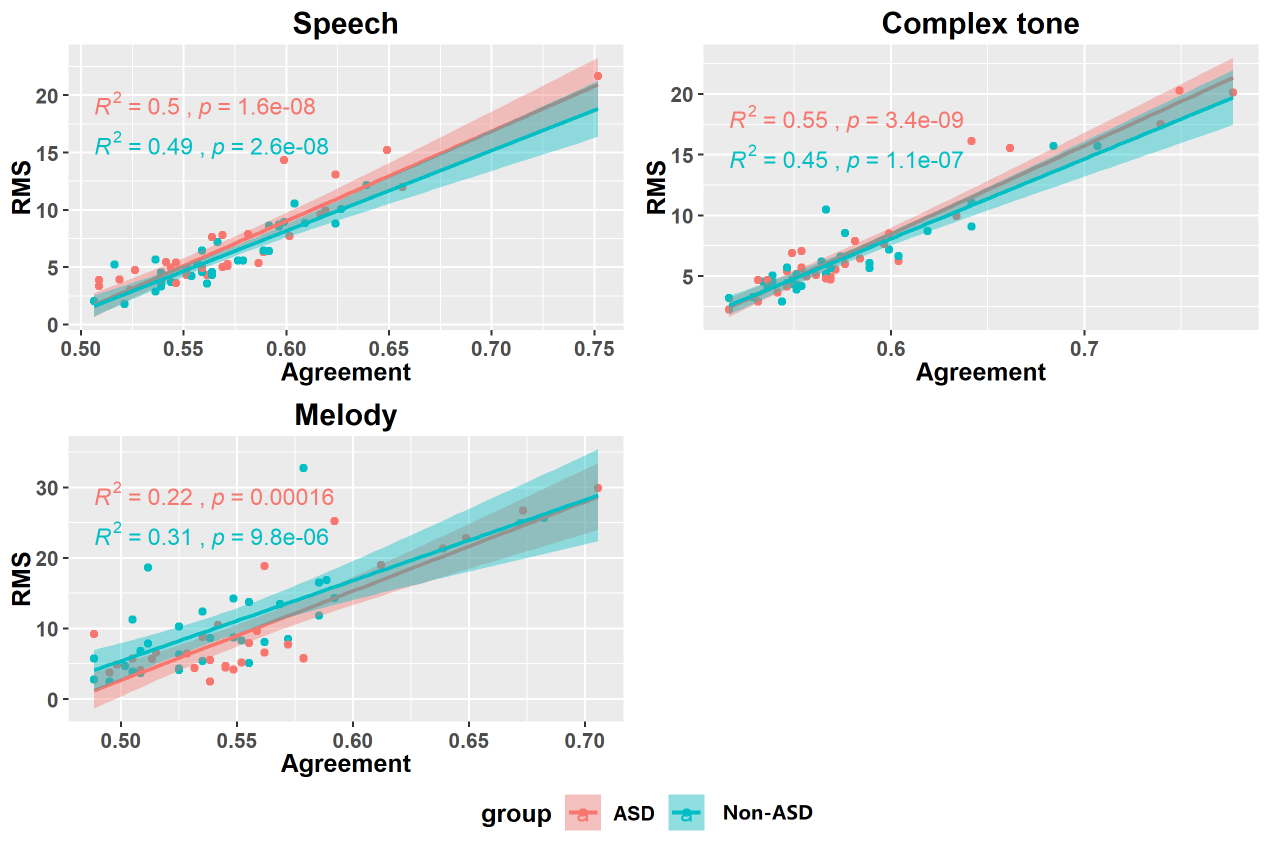


Figure S3. Scatter plots of ASD severity levels against performance on internal noise and RMS.


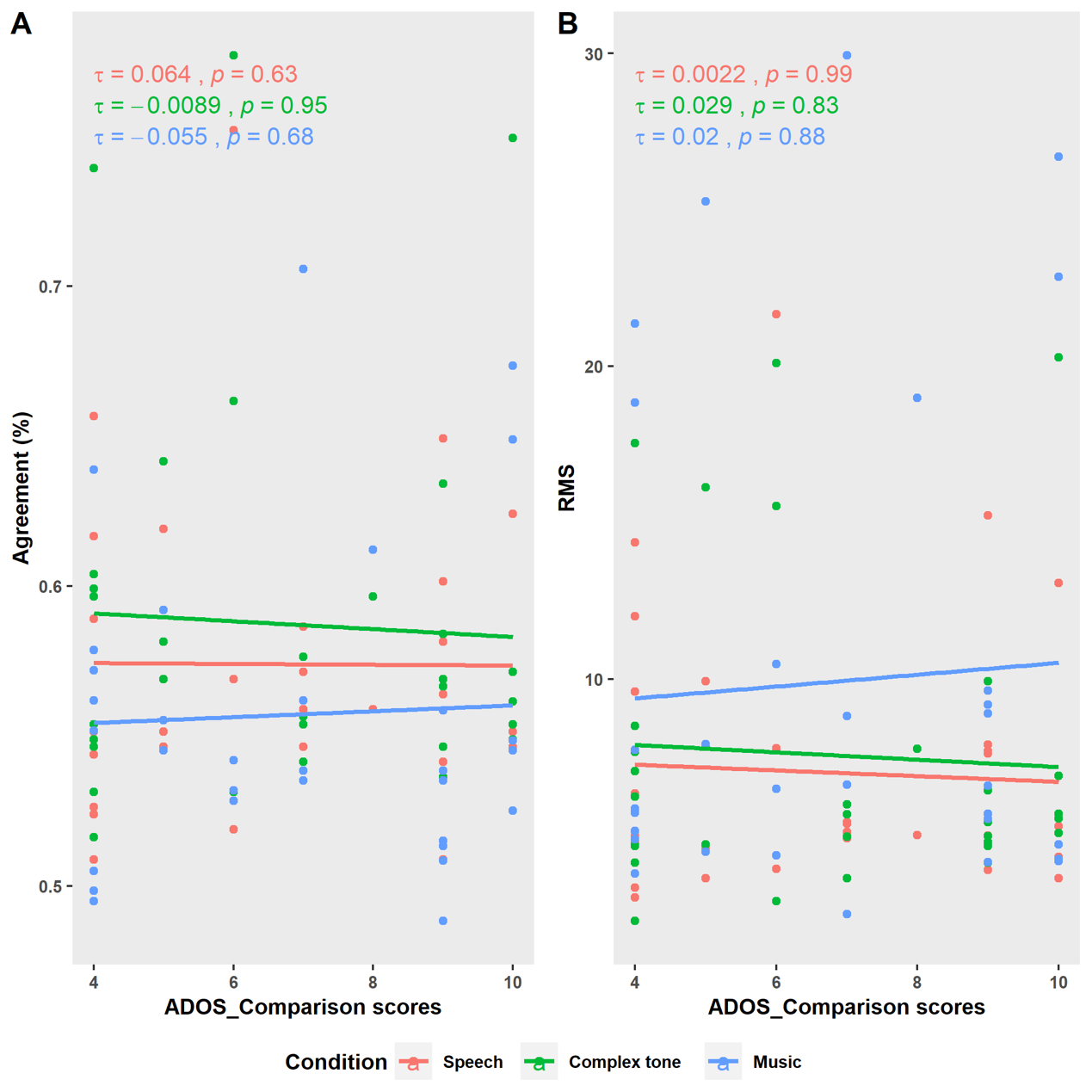


Table S1. Results of the linear mixed effects models across tasks, with the PPVT-R, gender, and age effects included.

| The speech vs. complex tone task | | | |
| --- | --- | --- | --- |
|  | df | F | *p* |
| Group | (1, 60) | 0.01 | 0.92 |
| Timepoint | (7,930) | 27.03 | < 0.001*** |
| Stimulus type | (1,936) | 32.69 | < 0.001*** |
| Group: Timepoint | (7,930) | 0.87 | 0.54 |
| Group: Stimulus type | (1.932) | 0.83 | 0.36 |
| Timepoint: Stimulus type | (7,930) | 3.82 | < 0.001*** |
| Group: Timepoint: Stimulus type | (7,930) | 0.14 | 0.99 |
| PPVT-R | **(1, 68)** | **0.01** | **0.91** |
| Gender | **(1,59)** | **0.38** | **0.54** |
| Age | **(1,59)** | **0.0006** | **0.98** |
| The melody task | | | |
|  | df | F | *p* |
| Group | (1, 59) | 1.91 | 0.17 |
| Note | (2,124) | 15.61 | < 0.001*** |
| Group: Note | (2,124) | 1.38 | 0.26 |
| PPVT-R | **(1, 59)** | **0.18** | **0.67** |
| Gender | **(1, 59)** | **0.36** | **0.55** |
| Age | **(1, 61)** | **0.16** | **0.69** |

*Note: *p < 0.05, **p < 0.01, and ***p < 0.001*

Table S2. The characteristics of participants who showed exceptional pitch sensitivity.

|  |  | Exceptional performance | | | Characteristics of the subgroup | | | |
| --- | --- | --- | --- | --- | --- | --- | --- | --- |
| ID | Group | Speech | Complex  tone | Melody | Age | Musical  training | RSPM | PPVT-R |
| Sub1 | ASD |  |  |  | 8.07 | 0 | 121.65 | 87 |
| Sub2 | ASD |  |  |  | 11.64 | 2 | 131.42 | 159 |
| Sub3 | ASD |  |  |  | 10.44 | 1 | 123.30 | 160 |
| Sub4 | ASD |  |  |  | 15.14 | 4 | 120.04 | 150 |
| Sub5 | ASD |  |  |  | 13.12 | 3 | 73.54 | 130 |
| Sub6 | ASD |  |  |  | 7.51 | 0.5 | 134.26 | 155 |
| Sub7 | ASD |  |  |  | 13.76 | 1 | 110.94 | 118 |
| Sub8 | ASD |  |  |  | 8.49 | 1.5 | 126.62 | 142 |
| Sub9 | Non-ASD |  |  |  | 10.3 | 0 | 112.33 | 141 |
| Sub10 | Non-ASD |  |  |  | 14.5 | 0 | 105.56 | 135 |
| Sub11 | Non-ASD |  |  |  | 14.94 | 0 | 0.57 | 127 |
| Sub12 | Non-ASD |  |  |  | 10.02 | 0 | 1.35 | 150 |

### **References**

Duda, M., Kosmicki, J. A., & Wall, D. P. (2014). Testing the accuracy of an observation-based classifier for rapid detection of autism risk. *Translational Psychiatry*, *4*(8), e424. https://doi.org/10.1038/tp.2014.65

Gotham, K., Pickles, A., & Lord, C. (2009). Standardizing ADOS Scores for a Measure of Severity in Autism Spectrum Disorders. *Journal of Autism and Developmental Disorders*, *39*(5), 693–705. https://doi.org/10.1007/s10803-008-0674-3
